# Protective Effect of Long-Term Fermented Soybeans with Abundant *Bacillus subtilis* on Glucose and Bone Metabolism and Memory Function in Ovariectomized Rats: Modulation of the Gut Microbiota

**DOI:** 10.3390/foods12152958

**Published:** 2023-08-04

**Authors:** Hee-Jong Yang, Ting Zhang, Yu Yue, Su-Ji Jeong, Myeong-Seon Ryu, Xuangao Wu, Chen Li, Do-Yeon Jeong, Sunmin Park

**Affiliations:** 1 Department of R & D, Microbial Institute for Fermentation Industry, Sunchang-gun 56048, Republic of Korea; godfiltss@naver.com (H.-J.Y.); yo217@naver.com (S.-J.J.); rms6223@naver.com (M.-S.R.); 2 Department of Bioconvergence, Hoseo University, Asan-si 31499, Republic of Korea; zhangting92925@gmail.com (T.Z.); niyani0@naver.com (X.W.); 3 Department of Food and Nutrition, Obesity/Diabetes Research Center, Hoseo University, Asan-si 31499, Republic of Korea; yuyue6491@gmail.com (Y.Y.); lic77732@gmail.com (C.L.)

**Keywords:** traditionally made doenjang, glucose metabolism, memory function, bone mineral density, estrogen deficiency

## Abstract

We investigated the effects of different types of long-term fermented soybeans (traditionally made doenjang; TMD) on glucose and bone metabolism and memory function in ovariectomized (OVX) rats. The rats were categorized into six groups: Control, cooked unfermented soybeans (CSB), and four TMDs based on *Bacillus subtilis* (*B. subtilis*) and biogenic amine contents analyzed previously: high *B. subtilis* (HS) and high biogenic amines (HA; HSHA), low *B. subtilis* (LS) and HA (LSHA), HS and low biogenic amines (LA; HSLA), and LS and LA (LSLA). The rats in the CSB and TMD groups fed orally had a 4% high-fat diet for 12 weeks. Rats in the Control (OVX rats) and Normal-control (Sham-operated rats) groups did not consume CSB or TMD, although macronutrient contents were the same in all groups. Uterine weight and serum 17β-estradiol concentrations were much lower in the Control than the Normal-control group, but CSB and TMD intake did not alter them regardless of *B. subtilis* and biogenic amine contents. HOMA-IR, a measure of insulin resistance, decreased with TMD with high *B. subtilis* (HSLA and HSHA) compared to the Control group. In OGTT and IPGTT, serum glucose concentrations at each time point were higher in the Control than in the Normal-control, and HSLA and HSHA lowered them. Memory function was preserved with HSHA and HSLA administration. Bone mineral density decline measured by DEXA analysis was prevented in the HSHA and HSLA groups. Bone metabolism changes were associated with decreased osteoclastic activity, parathyroid hormone levels, and osteoclastic activity-related parameters. Micro-CT results demonstrated that TMD, especially HSLA and HSHA, preserved bone structure in OVX rats. TMD also modulated the fecal bacterial community, increasing *Lactobacillus, Ligalactobacillus*, and *Bacillus*. In conclusion, through gut microbiota modulation, TMD, particularly with high *B. subtilis* content, acts as a synbiotic to benefit glucose, bone, and memory function in OVX rats. Further research is needed to make specific recommendations for *B. subtilis*-rich TMD for menopausal women.

## 1. Introduction

Menopause occurs when the ovaries stop producing estrogen. Estrogen is responsible for several functions in the body, such as regulating the menstrual cycle, controlling energy, glucose, and lipid levels, maintaining bone mineral density (BMD), and cognitive function [1,2]. Menopausal symptoms include hot flashes, mood swings, night sweats, and insomnia. In addition, menopause raises the risk of metabolic diseases along with obesity [1,2]. The incidence and severity of menopausal symptoms may vary depending on the geographic region and cultural factors. Asian women have a lower incidence of menopausal symptoms than Western women, which may be potentially related to differences in dietary patterns, such as a high intake of soybeans.

Estrogen promotes the production and upregulation of glucose transporters, especially glucose transporter-4 (GLUT-4), in cell membranes through estrogen receptors (ESR)-1 and ESR-2, allowing efficient glucose uptake from the bloodstream [3,4]. Estrogen deficiency, conversely, can induce insulin resistance and weight gain while also being a well-known factor contributing to BMD loss [5]. While higher body mass index, particularly in the overweight and obese range, is generally associated with increased mechanical loading on bones and stimulating bone formation, postmenopausal women often experience lower cortical bone mass and strength despite elevated insulin resistance [5,6,7]. Insulin resistance in prediabetic and diabetic patients is also associated with cognitive dysfunction, potentially through increasing Tau phosphorylation [8]. Interestingly, estrogen deficiency affects bone health and has implications for cognitive function [8]. Estrogen plays a role in brain connectivity, and its deficiency can contribute to cognitive impairment and amyloid-beta deposition, leading to Alzheimer’s disease development [9]. Low estrogen infusion to the brain enhances cognitive function in estrogen-deficient animal models [10]. Therefore, postmenopausal women with abdominal abnormal glucose metabolism are at a higher risk of experiencing osteoporosis and cognitive dysfunction.

Hormone replacement therapy with estrogen with or without progestogen improves menopausal symptoms. However, it may raise the risk of women-related cancers such as breast, ovarian, and uterine cancers. Alternative therapies involve natural remedies, dietary and lifestyle changes, herbal supplements, and other complementary medicines [11]. Phytoestrogens, mainly isoflavones, may protect against trabecular bone loss in postmenopausal women by enhancing bone formation and suppressing bone resorption [12]. Soybeans contain isoflavonoids known as phytoestrogens, and their intake is reported to improve bone density and skeletal muscle mass and reduce body weight after menopause [13]. However, fermented soybeans have isoflavone aglycones, which have greater bioavailability and may have better bone formation activity, and may improve cognitive function as observed in estrogen-deficient animal models [14,15,16]. Furthermore, some gut microbiota can convert daidzein in fermented soybeans, such as chungkookjang, into equol, the most potent phytoestrogen [17].

Traditionally made doenjang (TMD) is made by fermenting soybeans for about one year and is influenced by environmental factors, such as bacteria composition, fermentation temperature, seasonal variation, fermentation time, and salt contents [18]. TMD contains beneficial bacteria like *Bacillus*, *Lactobacillus*, *Pediococcus*, and *Weissella*, contributing to its potential as a synbiotic food [18]. However, some TMD products may also harbor low levels of harmful bacteria and contain biogenic amines during fermentation [18]. Biogenic amines are formed through the microbial decarboxylation of amino acids during fermentation. However, some *Bacillus subtilis* (*B. subtilis*) strains also have degradation activity of biogenic amines since different strains of bacteria have particular functions in fermented foods [19]. While TMD generally contains biogenic amines within safe levels (<200–500 mg/kg for fish and its products in CODEX) [20], it is essential to consider the varying quantities of these compounds in different TMD products. Excessive consumption of certain biogenic amines has been associated with adverse effects such as headaches, migraines, skin rashes, digestive issues, and allergic-like reactions [19]. However, the specific health implications of various biogenic amine quantities have not been extensively studied.

TMD intake improves the bioavailability of bioactive components in soybeans and menopausal symptoms in estrogen-deficient animal models [16]. It has also shown antiobesity activity in randomized clinical trials [21]. However, few studies have examined the TMD intake effect on glucose and bone metabolism and cognitive function in estrogen-deficient animal models. The present study aimed to investigate whether the TMD intake containing different quantities of *B. subtilis* and biogenic amines could improve glucose and bone metabolism and suppress cognitive impairment in OVX rats, known to be estrogen-deficient animal models.

## 2. Materials and Methods

### 2.1. Collection of TMD Products

Over 50 TMD products were purchased from different regions in Korea. Their characteristics were different due to varying annual temperatures, air pollution, and salt content. They were generally made by fermenting boiled soybeans with 10–13% salt for about 12 months outdoors [18,19]. Bacteria in TMD mainly originated from the air and were modulated by the salt content and bacterial community during fermentation [18,19]. Their compositions were analyzed for bacteria, biogenic amines, isoflavonoids, and sodium. The bacteria compositions in TMD were analyzed using the next-generation sequencing (NGS) in the 2.10 section based on previous studies [18,22].

The sodium content in TMD was quantified using ICP-AES (Thermo IRIS Intrepid II XDL, USA) following Korean MFDS guidelines. Protein digestion with nitric acid was performed prior to measurement. Methanol was mixed with TMD, and the filtrates were used to measure the sodium content. The oxygen and acetylene flows were set at 10.00 L/min and 2.50 L/min, respectively, with an air acetylene flame type, and the wavelength was set at 589.0 nm. Isoflavonoid glycone and aglycone contents in the filtrates were analyzed using HPLC (Agilent 1200 series, Agilent Technologies, Santa Clara, CA, USA) equipped with a Shiseido UG 120 column (4.6 × 250 mm, 5 μm, Osaka, Japan) according to the established procedure. As previously described, biogenic amine contents, including histamine and tyramine, were determined using HPLC analysis with a Cepcell Pak C18 column [23]. TMD was mixed with an internal standard and derivatized with sodium carbonate and dansyl chloride prior to measuring the biogenic amine contents. From the NGS results and biogenic amine contents of 50 TMD products, four TMD products were selected such as high and low *B. subtilis* at the cutoff of 70% of total bacteria and high and low biogenic amines at the cutoff of 300 mg/kg TMD. The four selected TMDs contained (1) High *B. subtilis* (HS) plus low biogenic amines (LA)(HSLA), (2) HS plus high biogenic amines (HA)(HSHA), (3) Low *B. subtilis* (LS) plus LA (LSLA), and (4) Low LS plus HA (LSHA).

### 2.2. Ovariectomy Procedure

Sixty female Sprague-Dawley rats (8 weeks old, weighing 163 ± 12 g) were obtained from DBL Co. Ltd. (Yeumsung-Kun, Republic of Korea) and individually housed in stainless-steel cages with a 12-h light/dark cycle at a room temperature of 23 °C. Following a week of acclimatization at the animal facility of Hoseo University, the rats underwent ovariectomy (OVX) surgery. An animal study was performed under the ethical guidelines and regulations, approved by the Hoseo University Animal Care and Use Committee (Approval No. HSIACUC-22-03), and aligned with the principles outlined in the Guide for the Care and Use of Laboratory Animals by the National Institutes of Health (NIH) in the USA. Both ovaries of sixty rats were dissected with scissors after subcutaneously injecting a ketamine/xylazine mixture (100 and 10 mg/kg body weight). An additional ten rats underwent a sham operation for ovariectomy. 

### 2.3. Diet Preparation

All the groups were given a baseline high-fat diet (HFD), which diminished bone mineral density and bone microstructure [6]. This diet was formulated to contain 43.4%, 17.1%, and 39.5% of energy (En%), fat, protein, and carbohydrates, respectively, and 5.9 g salt/kg based on the AIN-93 diet for small animals [24]. The diet was supplemented with either 4% (*w/w*) of lyophilized doenjang or 4% (*w/w*) of cooked soybeans. As doenjang and soybeans contain fats, proteins, and carbohydrates, the nutrient composition of these ingredients was subtracted from the corresponding components in the corresponding diet to achieve uniform carbohydrate, fat, and protein composition across all diets. This adjustment ensured that all diets had equivalent carbohydrate, fat, and protein composition, allowing for a fair comparison of the effects of the interventions. The assigned starch, casein, vitamins, minerals, and either TMD or cooked soybeans were combined and mixed. Subsequently, lard and soybean oil were added to the mixture, and the ingredients were sifted to remove lumps. All the diets in the TMD and control groups had equivalent amounts of carbohydrates, proteins, fats, and sodium.

### 2.4. Experimental Design

Sixty OVX rats were randomly allocated to each group and given either cooked soybeans or four different TMD products. Each group was labeled based on the type of diet administered: (1) Control (high-fat diet without TMD or cooked soybeans), (2) cooked soybeans (CSB), lyophilized TMD with (3) HSHA, (4) HSLA, (5) LSHA, and (6) LSLA. In addition, ten sham-operated rats were included as Normal controls. All the diets had an equivalent macronutrient composition. Throughout the twelve-week study period, all rats were provided unrestricted access to food and water, and their food intake and body weight were monitored and recorded weekly.

### 2.5. Glucose Metabolism Measurement

After the 11-week TMD intervention, the animals underwent an oral glucose tolerance test (OGTT) following an overnight fasting period. During the test, they were orally administered 2 g of glucose per kg of body weight. Tail blood samples were collected at 10-min intervals for up to 120 min to measure serum glucose concentrations. Additionally, serum insulin concentrations were assessed at 0, 20, 40, 90, and 120 min. The trapezoidal rule was applied to calculate the mean area under the curve (AUC) for both serum glucose and insulin concentrations. Three days after the OGTT, the animals underwent an intraperitoneal insulin tolerance test (IPITT) following a 6-h fast. Their serum glucose levels were measured at 15-min intervals for 90 min after receiving an intraperitoneal insulin injection at a dosage of 0.75 U/kg of body weight.

### 2.6. Memory Function Measurement

A passive avoidance test was conducted using a shuttle box apparatus equipped with two dark/light compartments [25]. A rat was initially placed in the light compartment. When it entered the dark chamber, electrostimulation (75 V, 0.2 mA, 50 Hz) was delivered in two acquisition trials with an 8-h interval. In the third trial, the latency taken to enter the dark chamber was assessed without electrostimulation for 600 s. A longer latency indicates better memory function.

For the forced swimming test, the rat was food-deprived for 8 h and then subjected to a 10-min pretest in a clear acrylic cylinder. The cylinder was 60 cm in height and 30 cm in width, and it was filled with 45 cm of water maintained at a temperature of 24 ± 1 °C. After 24 h, a 5-min forced swimming test was conducted in the same cylinder. During this test, the rat’s movements were recorded and scored for mobile behaviors, such as swimming and climbing, as well as immobile behaviors [25]. The total time spent on active and passive behaviors was calculated. Rats showing longer active time during the forced swimming session were considered less depressed.

The novel object recognition test was performed by modifying previously established methods [25]. On the first two days, rats were given 5 min to explore a plexiglass box. During the training phase, the box was divided into four areas, and two identical objects were placed in the center of the diagonal area. Rats were then placed in the center of the box and allowed to explore the objects for 5 min freely. Afterward, the objects and instruments were cleaned with 75% ethanol to remove residual odor. On the final day of the experiment, one of the objects was replaced with a new object that was similar in size but differed in shape and color from the original object. The rats’ exploration behavior, which included nose/paw contact (excluding sitting on or turning the object around), was recorded for both the old and new objects. The time spent exploring each object was measured. The recognition index was calculated by dividing the time spent exploring the new object by the total time spent exploring both objects. If the recognition index was lower than 50%, it was considered incidental, indicating poor recognition. Higher values of the recognition index indicated good recognition of the new object.

### 2.7. BMD Measurement by Dual-Energy X-ray Absorptiometry (DEXA) and Sample Collection

Two days after the IPITT, the body composition of the rats was assessed using DEXA while they were under anesthesia with a ketamine/xylazine mixture. The DEXA measurements were performed using a DEXA machine supplied by Norland Medical Systems Inc., Fort Atkinson, WI, USA. Before and after the experiments, a calibration phantom was used to ensure accurate measurements of the rats’ body composition. The anesthetized rat was positioned in a prone position with its rear legs securely in external rotation, and the hip, knee, and ankle joints were flexed at 90° for the DEXA scan. Following the scan, the BMD was determined using the DEXA machine’s software designed explicitly for rodent BMD analysis [26].

After completing the DEXA analysis, the rats were euthanized. While under anesthesia induced by a combination of ketamine and xylazine, blood samples were drawn from the inferior vena cava. The femur was then carefully dissected following the 12-week treatment period. Next, the epididymal and retroperitoneal fat masses were excised and weighed, along with the uteri. The uterus index was calculated by dividing the uterus weight by the body weight of the rats. Blood was collected via cardiac puncture and then centrifuged at 3000 rpm for 20 min to obtain serum samples. Additionally, tissues and fecal samples were collected and carefully preserved at −70 °C for future analyses.

### 2.8. Micro-Computed Tomography (CT) of the Femur

The femur was then fixed with 4% paraformaldehyde, and the BMD was determined in vivo through X-Ray Radiography Micro-computed tomography (Micro-CT; Skyscan 1273, Billerica, MA, USA) in Korea Basic Science Institute, Aging Science Center (Kwangju, Republic of Korea).

### 2.9. Biochemical Assay

The homeostasis model assessment for insulin resistance (HOMA-IR) was determined by applying the following formula: fasting insulin (µIU/mL) × fasting glucose (mM)/22.5. The Glucose Analyzer II (Beckman-Coulter, Palo Alto, CA, USA) and Ultrasensitive insulin ELISA kits (R&D Diagnostics, Minneapolis, MN, USA) were employed to measure the serum glucose and insulin concentrations, respectively. ELISA kits from Enzo Life Sciences (Farmingdale, NY, USA) were employed to assess serum 17β-estradiol levels. Additionally, BMD-related biomarkers in circulation were measured using ELISA kits. These biomarkers included rat osteoprotegerin (OPG; Abcam, Cambridge, UK), receptor activator of nuclear factors-κB ligand (RANKL; Abcam), osteocalcin (Abcam), parathyroid hormone (PTH; Abcam), and bone-specific alkaline phosphatase (BALP; MyBioSource, San Diego, CA, USA).

Serum was collected from portal vein blood and combined with acidic ethanol (0.01 N HCl; Duksan, Republic of Korea). SCFA concentrations in the resulting supernatants were measured using a gas chromatograph (Clarus 680 GAS, PerkinElmer, Waltham, MA, USA) equipped with an Elite-FFAP capillary column (30 m × 0.25 mm × 0.25 μm) [27]. External standards of 1 mM acetate, propionate, and butyrate (Sigma Co., St. Louis, MO, USA) were used for calibration.

### 2.10. Gut Microbiota in the TMD and Feces Evaluated by NGS

Metagenome sequencing using NGS techniques was conducted to analyze the microbial communities in TMD and fecal samples obtained from the cecum [10]. Bacterial DNA from the feces was extracted using the Power Water DNA Isolation Kit (Qiagen, Valencia, CA, USA). PCR products were amplified with 16S amplicon primers in the FastStart High-Fidelity PCR System (Roche, Basel, Switzerland), following the GS FLX plus library prep guide [10]. The bacterial DNA in the feces was then sequenced using Illumina MiSeq and a Genome Sequencer FLX plus 454 Life Sciences (Illumina; San Diego, CA, USA) following the manufacturer’s instructions in Macrogen (Seoul, Republic of Korea) [28]. 

The 16S amplicon sequences were processed using Mothur v.1.36, and the Miseq standard operation procedure was applied to identify the taxonomy of fecal bacteria. Silva reference alignment v.12350 was utilized to align the sequences, and relative bacterial counts were determined based on taxonomic assignment for each sample [28]. The sequences classified as mitochondria, Eukaryota, or unknown were removed. Operational taxonomic units (OTUs) below 10,000 reads were deleted. The principal coordinate analysis (PCoA) outcomes of gut microbiota were visualized using the R package, as described previously [10].

Metagenome function analysis was conducted using the PICRUSt2 software to investigate the differences among gut microbiota in the various groups. The metabolic functions of the fecal bacteria were predicted from the FASTA files and counted using tables PICRUSt2, and these predictions were based on the Kyoto Encyclopedia of Genes and Genomes (KEGG) Orthologues (KO) mapped through the KEGG mapper (https://www.genome.jp/kegg/tool/mapper/search.html, accessed on 23 March 2023), as previously described [10].

### 2.11. Statistical Analysis

Statistical analysis was performed using SAS software version 7 (SAS Institute, Cary, NC, USA). The optimal sample size of 10 per group was determined using the G power program with a power of 0.90 and an effect size of 0.5. After confirming the normal distribution through Proc univariate, the data were expressed as mean ± standard deviation (SD). One-way ANOVA was employed to analyze the measurements, and Tukey’s test was used to assess differences among the groups. Statistical significance was considered at *p* < 0.05.

## 3. Results

### 3.1. The Characteristics of Dried TMD with Different Amounts of B. subtilis and Biogenic Amines

Water content in the TMD samples was around 50% (50–59%), and the dried TMD contained approximately 7–9% salts (Table 1). The amounts of histamine and tyramine, biogenic amines, were higher in two TMDs (HSHA and LSHA) than in the others (Table 1). The biogenic amine contents of HSHA and LSHA were higher than HSLA and LSLA. The bacterial composition of the TMD products, as determined by NGS, exhibited that two HSHA and HSLA contained about 90% *B. subtilis* but other TMDs (LSHA and LSLA) were composed of less than 60%. LSHA and LSLA contained about 20 and 35% of *B. licheniformis*. *Leuconostoc mesenteroides*, *Leuconostoc citreum*, *Staphylococcus aureus*, and *Acinetobacter baumannii*, potentially harmful bacteria, were less than 1% in all TMDs (Table 1).

CSB contained high in isoflavonoid glycans and low in isoflavonoid glycans. After fermentation, isoflavonoid glycans were not detected in LSHA, HSHA, and HSLA, but LSLA contained a small number of isoflavonoid glycans. However, TMD included much higher amounts of isoflavonoid aglycans than CSB. Among TMDs, LSLA contained less isoflavonoid aglycon than LSHA, HSHA, and HSLA (Table 1). Total isoflavonoids were much lower in TMDs than CSB, and LSLA contained them the lowest.

### 3.2. Energy and Glucose Metabolism

Throughout the 12-week intervention, the Control group displayed a higher body weight increase than the Normal-control group. In contrast, all TMD groups exhibited lower weight gain than the Control group, but the reduction was not as significant as observed in the Normal-control group (Table 2). CSB did not result in decreased weight gain compared to the Control group. However, it is noteworthy that all groups showed no significant difference in food intake (Table 2).

The Control group exhibited significantly lower serum 17β-estradiol concentrations and uterine weight than the Normal-control group. The administration of TMD or CSB did not affect these concentrations (Table 2). Uterine weight was notably higher in the Normal-control group than in the Control group, but TMD did not significantly impact it (Table 2). The fasting and 2-h post-prandial serum glucose concentrations were higher in the Control group than in the Normal-control group. HSLA prevented this increase. However, the 2-h post-prandial serum glucose concentrations in the HSHA and HSLA groups were similar to those of the Normal-control group (Table 2). Fasting serum insulin concentrations were higher in the Control group compared to the Normal-control group. Interestingly, HSHA, HSLA, and CSB intake led to decreased insulin concentrations. The serum insulin concentrations in the HSLA and CSB groups were similar to those of the Normal-control group (Table 2). HOMA-IR, an insulin resistance index, was significantly higher in the Control group than in the Normal-control group. However, HSHA, HSLA, and CSB intake resulted in similar levels of HOMA-IR compared to the Normal-control group (Table 2).

### 3.3. OGTT and IPITT

**Figure 1:**
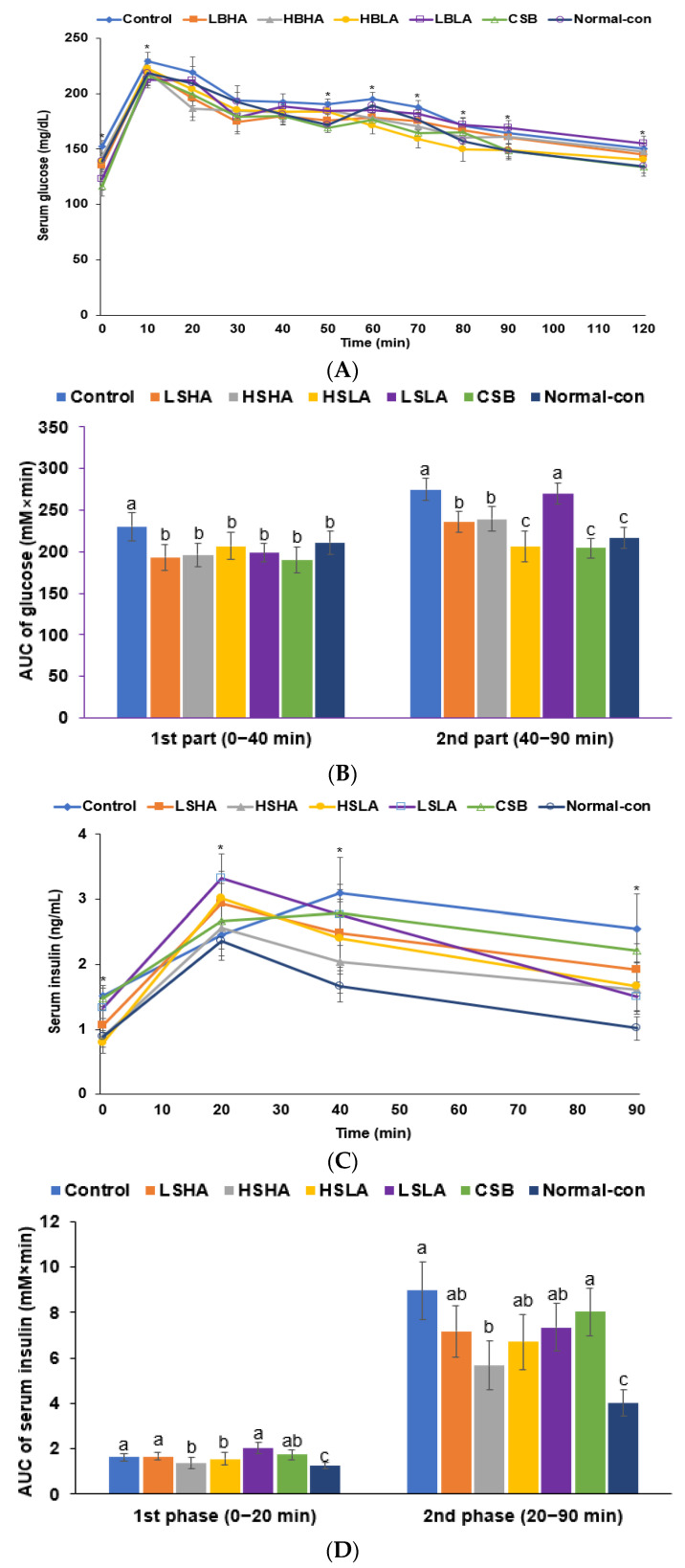
Serum glucose and insulin concentrations after the oral intake of 2 g glucose per kg body weight during oral glucose tolerance test (OGTT). (**A**) Changes in serum glucose concentrations. (**B**) The area under the curve (AUC) of serum glucose concentrations during the OGTT. (**C**) Changes in serum insulin concentrations. (**D**) The AUC of serum insulin concentration during the OGTT. Dots or bars and error bars represent the means ± standard deviations (*n* = 10). * significantly different among six groups at *p* < 0.05. ^a,b,c^ Different letters on the bars indicate a significant difference among the groups by Tukey’s test at *p* < 0.05.

Following the administration of 2 g of glucose per kilogram of body weight in the OGTT, the serum glucose concentrations of all rats steadily increased up to 30–40 min and then gradually decreased (Figure 1A). At 20 min, the peak serum glucose concentrations did not show significant differences between the groups, but they markedly decreased between 30 and 50 min. Subsequently, the concentrations gradually decreased from 50 min onwards. The serum glucose concentrations in the Control group were higher than those in the Normal-control group, whereas they were lower in the LSHA and CSB groups. The AUC of the serum glucose concentrations from 0–40 min was higher in the Control group than the Normal-control group, while it was lower in all the TMD and CSB groups than in the Normal-control (Figure 1B). However, the AUC from 40–90 min was much higher in the Control group than the Normal-control group, while the AUC in the LSHA and CSB groups was similar to that of the Normal-control (Figure 1B).

During the OGTT, serum insulin concentrations in the Control group increased to 30 min but peaked at 20 min for the other groups. The Normal-control group showed the lowest serum insulin concentrations, with the HSHA and HSLA groups displaying levels similar to the Normal-control (Figure 1C). The AUC of the serum insulin concentrations during 0–20 min was higher in the Control group compared to the Normal-control group, and it was lower in the HSHA and HSLA groups than the Control group but higher than the Normal-control (Figure 1D). The AUC of the serum insulin concentrations from 20 to 90 min was much higher in the Control group compared to the Normal-control group. While it was lower in the HSHA group than the Control group, it was not as much as in the Normal-control group (Figure 1D).

**Figure 2:**
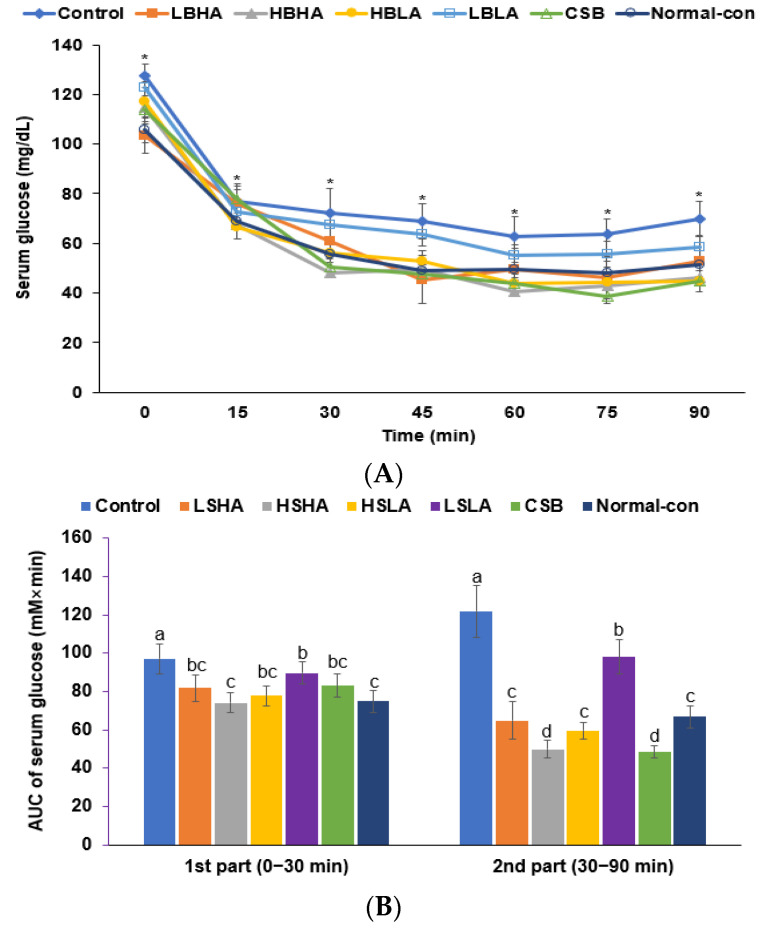
Serum glucose concentrations after the intraperitoneal injection of 1 U insulin per kg body weight during intraperitoneal insulin tolerance test (IPITT). (**A**) Changes in the serum glucose concentrations. (**B**) The area under the curve (AUC) of serum glucose concentrations during the IPITT. Dots or bars and error bars represent the means ± standard deviations (*n* = 10). * significantly different among six groups at *p* < 0.05. ^a,b,c,d^ Different letters on the bars indicated a significant difference among the groups by Tukey’s test at *p* < 0.05.

Following intraperitoneal insulin injection after 6-h food deprivation in the IPITT, serum glucose concentrations decreased in all rats. However, the decrease was less significant in the Control group than in the other groups (Figure 2A). At 60–90 min, the concentrations in the HSHA, HSLA, and CSB groups were lower than in the Normal-control. The AUC of the serum glucose concentrations was much higher in the Control than in the Normal-control group (Figure 2A). Meanwhile, the AUC for the HSHA, HSLA, LSLA, and CSB groups decreased during the 0–30 min period. The AUC between 30 and 90 min also showed a similar decrease but to a greater extent (Figure 2B). This indicates that HSHA, HSLA, and CSB can improve glucose homeostasis and reduce insulin resistance.

### 3.4. Memory Functions and Depression

**Figure 3:**
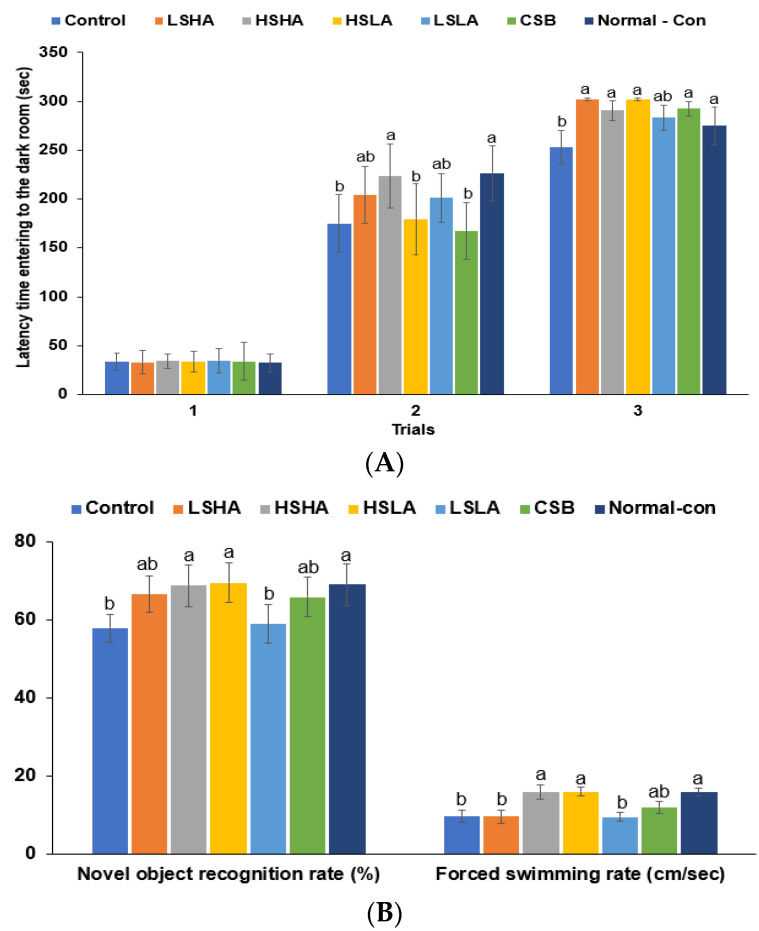
Cognitive function. (**A**) The latency in entering the dark room in the passive avoidance test. (**B**) Novel object recognition rate and forced swimming test by rats. Bars and error bars represent the means ± standard deviations (*n* = 10). ^a,b^ Different letters on the bars indicated a significant difference among the groups by Tukey’s test at *p* < 0.05.

In the passive avoidance test, the latency in entering the dark room was significantly shorter in the Control group compared to the Normal-control group in the second trial, and it was only extended in the HSHA group. In the third trial, the latency was longer in all the TMD and CSB groups except LSLA, and it was similar to that of the Normal-control group (Figure 3A). The novel object recognition rate was lower in the Control group than in the Normal-control group during the novel object recognition test. However, administration of HSHA and HSLA to OVX rats increased the recognition rate, making it comparable to that of the Normal-control group (Figure 3B). The swimming rate assessed depression during the forced swimming test (Figure 3B). The forced swimming rate was lower in the Control group than the Normal-control group but increased in the HSHA and HSLA groups, reaching levels similar to that of the Normal-control group. The result suggests that OVX rats induced depression, and HSHA and HSLA treatments effectively ameliorated it, similar to the Normal-control group.

### 3.5. BMD by DEXA and Micro-CT

**Figure 4:**
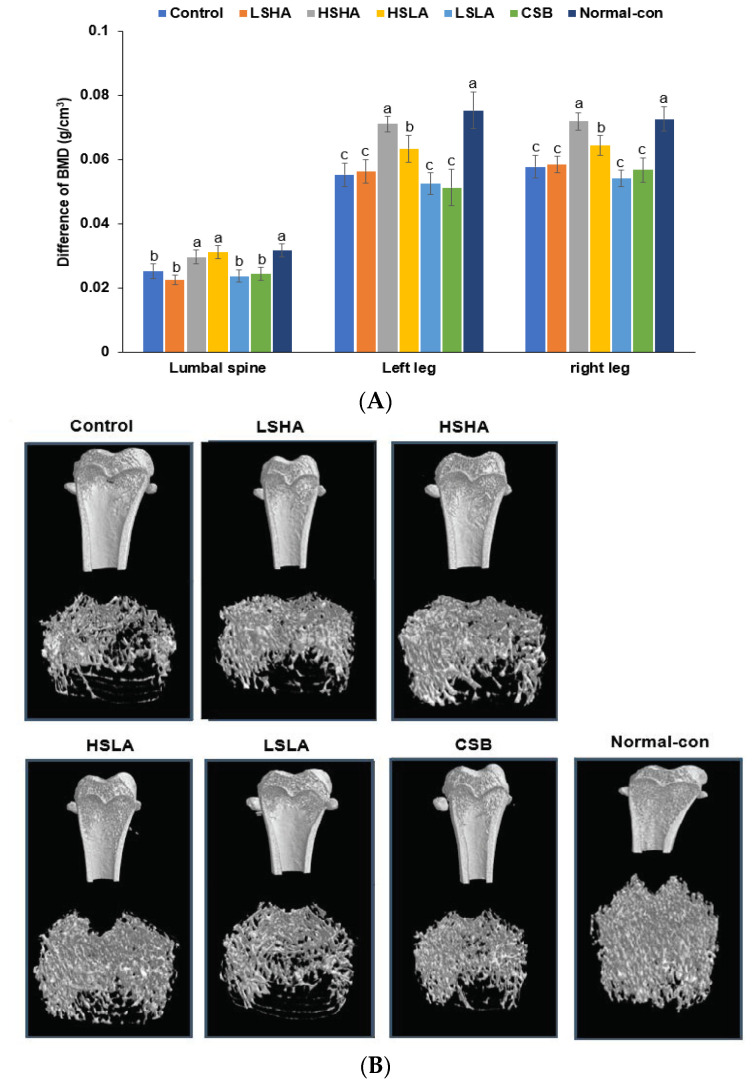
Bone mineral density measured by dual X-ray absorptiometry (DEXA) and Micro-computed tomography (CT). (**A**) Differences in the BMD before and after the 12-week intervention of TMD or CSB. (**B**) Image of micro-CT analysis in the distal femur after the 12-week treatment. Bars and error bars represent the means ± standard deviations (*n* = 10). ^a,b,c^ Different letters on the bars indicate a significant difference among the groups by Tukey’s test at *p* < 0.05.

The DEXA analysis showed that the differences in the BMD of the lumbar spine and left and right legs between the pre- and post-interventions were lower in the Control group than in the Normal-control group based on measurements taken before and after the 12-week intervention (Figure 4A). In the HSHA and HSLA groups, there was no difference in the BMD of the lumbar spine after the interventions, similar to the Normal-control. The differences in BMD of the legs were higher than that of the lumbar spine, and the decrease in the HSHA group was similar to that of the Normal-control (Figure 4A). The difference in the BMD of the legs was lower in HSLA than in the Normal-control.

At the end of the 12-week intervention, the Micro-CT analysis revealed that the BMD in the Control group was significantly lower than in the Normal-control group (Figure 4B). However, all the TMD and CSB groups showed significantly higher BMD than the Control group. The HSLA group exhibited a remarkable prevention of BMD loss, although its BMD levels did not fully reach those observed in the Normal-control (Table 3, Figure 4B). The ratio of segmented bone volume to the total volume of the bone region (BV/TV) was higher in the Normal-control compared to the Control, and the TMD and CSB treatments effectively prevented the decrease in BV/TV. The HSLA group showed the highest BV/TV, though it still remained lower than the Normal-control (Table 3; Figure 4B). The mean trabecular thickness (Tb_Th) exhibited a similar trend to BV/TV (Table 3; Figure 4B). Moreover, the average trabecular number (Tb.N) was lower in the Control group than in the Normal-control group, and the HSLA group showed the highest Tb.N among the TMD and CSB treatment groups. On the other hand, the mean trabecular distance (Tb.Sp) displayed an opposite trend to that of Tb.N (Table 3; Figure 4B). Overall, both HSLA and HSHA treatments were effective in partially preventing BMD loss better than CSB in estrogen deficiency-induced conditions. However, they were unable to maintain BMD at the level of the Normal-control group fully.

The serum PTH concentrations were notably higher in the Control group than in the Normal-control group (Table 4). However, the administration of CSB and TMD, except for LSHA and LSLA, resulted in decreased serum PTH concentrations compared to the Control group. Remarkably, HSHA administration led to a lower concentration of PTH, even comparable to the levels observed in the Normal-control group. Regarding the serum RANKL concentrations, they increased in the Control group when compared to the Normal-control group. However, the administration of TMD and CSB, except for LSHA and LSLA, decreased RANKL concentrations, bringing them to similar levels as in the Normal-control group (Table 4). As for the serum OPG concentrations, they were reduced in the Control group when compared to the Normal-control group. Nevertheless, the administration of HSHA, HSLA, and CSB effectively decreased the OPG levels, bringing them to levels similar to those observed in the Normal-control group (Table 4). Moreover, the serum osteocalcin concentrations were higher in the Control group than in the Normal-control group, but the administration of HSHA, HSLA, and CSB successfully prevented their increase (Table 4). As an index of bone turnover rate, serum BALP concentrations were higher in the Control group than in the Normal-control group. The administration of TMD and CSB prevented the increase in BALP concentrations. However, only the CSB group exhibited BALP concentrations similar to those in the Normal-control group (Table 4).

### 3.6. SCFA in the Blood from the Portal Vein and Fecal Bacteria

**Figure 5:**
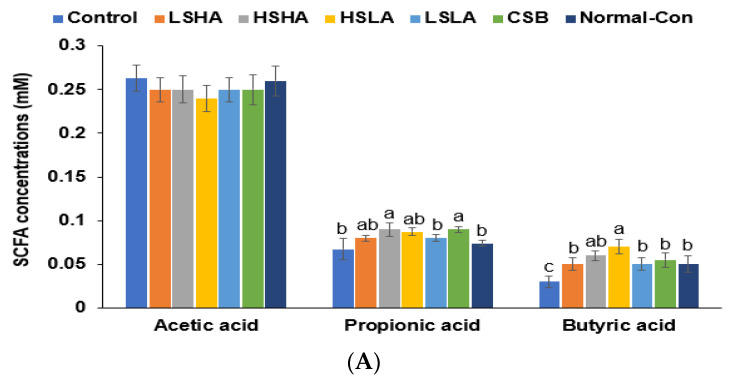

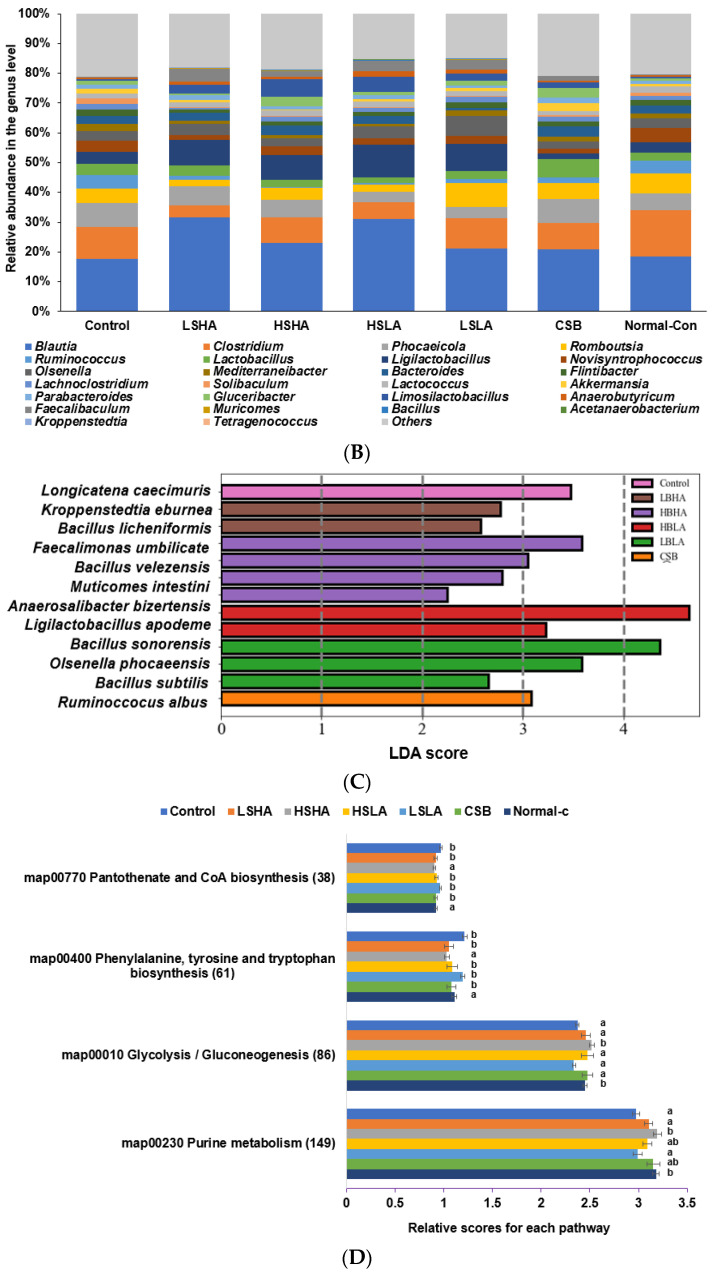

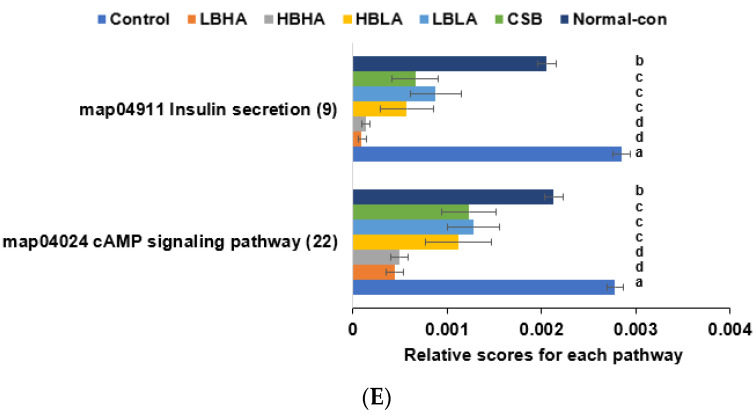
Fecal bacterial analysis after the 12-week intervention. (**A**) The short-chain fatty acid concentration in the portal vein. (**B**) Relative abundance of fecal bacteria from the cecum at the genus level. (**C**) Linear discriminant analysis (LDA) score. (**D**) Metagenome analysis in glucose, amino acid, and purine metabolism. (**E**) Metagenome analysis in insulin secretion and cyclic adenosine 3′,5′-monophosphate (cAMP) signaling pathways. Bars and error bars represent the means ± standard deviations (*n* = 10). ^a,b,c,d^ Different letters on the bars indicated a significant difference among the groups by Tukey’s test at *p* < 0.05.

The levels of acetate and propionate in the portal vein did not show significant differences among all the groups (Figure 5A). However, butyrate concentrations were notably lower in the Control group compared to the Normal-control group. The HSLA and CSB groups exhibited the highest butyrate levels among the intervention groups, whereas the LSHA and LSLA groups had relatively lower levels (Figure 5A).

The Control group had somewhat different fecal bacteria than the Normal-control and the TMD intake altered their composition (Figure 5B). The levels of the genus *Blautia* were higher in the LSHA and HSLA groups than the other groups, and levels of the genus *Bacillus* were higher in the TMD groups than in the Control and Normal-control groups (Figure 5B). The TMD intake increased the abundance of the genus *Lactobacillus* and *Ligilactobacillus* compared to the Control and Normal-control groups, and the intake of LSLA increased the abundance of the genus *Olsenella* compared to the other groups (Figure 5B). Although the α-diversity did not vary among the groups, TMD was separated from the Control in the β-diversity (*p* < 0.001). In linear discriminant analysis (LDA) analysis, *Bacillus* species were selected as the primary bacteria in the TMD but not the CSB group. *Ligilactobacillus apodemi* had a high LDA level in the HSLA group, and *Olsenella phocaeensis* and *Clostridium hylemonae* were high in the LSHA group (Figure 5C). Regarding metagenome function analysis, glycolysis/gluconeogenesis and purine metabolism were lower in the Control group compared to the Normal-control group. CSB and TMD, except LSLA, prevented the decrease in these functions. Pantothenate and coenzyme A (CoA) biosynthesis and phenylalanine/tyrosine/tryptophan biosynthesis showed an opposite trend to glycolysis/gluconeogenesis in fecal bacteria (Figure 5D). Insulin secretion and the cyclic adenosine 3′,5′-monophosphate (cAMP) signaling pathways were higher in the Control group compared to the Normal-control group. TMD and CSB prevented an increase in the signaling of these pathways (Figure 5E).

## 4. Discussion

TMD has been reported to ameliorate obesity, hypertension, neuroinflammation, neurodegeneration, and oxidative stress [22]. However, few studies have been reported to evaluate TMD effects in improving estrogen-deficiency-associated symptoms, glucose, energy, and bone metabolism [29]. We aimed to investigate how different TMD types modulate glucose, bone metabolism, and memory function in OVX rats, an estrogen-deficient animal model. The novel findings of the current study are as follows. The present study showed that TMD decreased serum glucose concentrations during OGTT and improved insulin resistance, which helped prevent glucose-induced bone loss and memory dysfunction. The HSHA and HSLA types of TMD were particularly effective in preventing the reduction in BMD and memory function. However, the study also found that the abundance of *Bacillus* in TMD may affect its efficacy for glucose metabolism, bone density, and memory function. Further research is needed to evaluate the optimal TMD type to achieve the abovementioned benefits.

Furthermore, fermented soybeans such as chungkookjang and TMD improve glucose metabolism [30]. Both contain isoflavone aglycones more than isoflavone glycones compared to unfermented soybeans, and the process to remove glucose from the isoflavone glycones is conducted with *Bacillus* species, particularly *B. subtilis*, *B. amyloliquefaciens*, and *B. licheniformis* in chungkookjang and TMD. Therefore, adults showing a better response to soybeans may have specific intestinal microbes (e.g., *Lactobacillus intestinalis* and *Lactobacillus johnsonii*) that convert daidzein into equol, a potent phytoestrogen, potentially to alleviate estrogen-deficient symptoms [17]. These intestinal microbes exist in those consuming soybean products, especially fermented soybeans. The present study showed that all TMD types decreased glucose intolerance, mainly by enhancing insulin sensitivity. Therefore, TMD can improve glucose tolerance and insulin resistance, and among the various TMD types, HSLA was most effective in regulating glucose homeostasis by enhancing insulin sensitivity in estrogen-deficient rats. Isoflavonoids in HSLA might have a higher chance of being converted into equol, which is potent for preventing and ameliorating BMD loss. It needs to have further study. 

There is a close relationship between glucose and bone metabolism and cognitive function, and estrogen deficiency adversely affects all three parameters. Osteoporosis has been linked to cognitive decline and memory impairment, likely due to blood flow changes and brain oxygen delivery [31]. Consistent with previous studies [31], in the present study, an increase in serum PTH concentrations was observed in estrogen-deficient rats, which increased bone resorption, leading to decreased BMD as measured by the DEXA and micro-CT methods and elevated bone resorption indexes such as RANKL, osteocalcin, and BALP. HSLA and HSHA prevented the decrement of BMD compared to the Control group. CSB declined bone resorption compared to the Control group, but the HSLA and HSHA groups exhibited lower bone resorption than the CSB group. Although the relationship between soybean intake and BMD remains controversial in estrogen-deficient states, dietary isoflavonoids attenuate menopause-induced osteoporotic bone loss by decreasing bone resorption and stimulating bone formation [32]. Isoflavonoids decrease bone resorption through the OPG/RANKL/RANK pathways, similar to the HSLA and HSHA activity observed in the present study [31]. Previous research has shown that consuming fermented soybean products like natto and doenjang is associated with increased BMD in postmenopausal women [33,34,35]. In the current study, the intake of HSLA and HSHA, among various types of TMDs, effectively prevented BMD loss in OVX rats by reducing bone resorption by decreasing serum PTH concentration. These findings strongly suggest that the presence of high B. subtilis in TMD plays a critical role in mitigating BMD loss. This observation aligns with other studies that have highlighted the positive impact of B. subtilis C-3102 on BMD improvement in postmenopausal women through mechanisms such as inhibiting bone resorption, enhancing fecal Bifidobacterium, and reducing Fusobacterium [36]. Therefore, HSLA and HSHA act as synbiotics to improve BMD in an estrogen-deficient state.

The decrease in glucose metabolism with insulin resistance contributes to reduced synaptic plasticity [37], suggesting brain insulin resistance leads to cognitive decline. Estrogen deficiency is also involved in exacerbating insulin resistance in the brain, possibly disturbing cognitive function [38]. Postmenopausal women who consume soy isoflavones have better cognitive function compared to those who do not consume soybeans [38]. Both unfermented and fermented soybeans (chungkookjang and doenjang) improve memory function in an estrogen-deficient state [16,39,40,41]. However, long-term fermented soy products may have a greater effect on improving cognitive dysfunction, particularly memory, due to *Bacillus* species and changes in the soybean components, such as increased isoflavone aglycones, as observed in previous studies [40,41]. The present study has demonstrated that the memory function assessed using passive avoidance and novel object recognition tests was improved in HSLA and HSHA-treated estrogen-deficient rats compared to the Control group. The improvement in HSLA and HSHA-treated rats was greater than in CSB-treated rats. HSLA and HSHA contain an abundance of *Bacillus* species, primarily *B. subtilis*, *B. coagulans*, *B. amyloliquefaciens*, *and B. velezensis* that produce short-chain fatty acids, especially butyrate and propionate, similar to chungkookjang [39,40,41]. Therefore, better memory function in fermented soybeans is potentially related to the *Bacillus* species and their metabolites.

The composition of fecal bacteria can exhibit variations compared to that of the gut, as observed in previous studies [42]. Factors such as transit time through the gut, interactions with host cells and other bacteria, and environmental influences contribute to these differences in fecal bacterial composition. Furthermore, the abundance of bacterial groups in feces is known to be influenced by factors such as diet, age, and health status [42,43]. However, whether specific bacterial species are more likely to be excreted in the feces is still unclear. In our present study, we found that an increased intake of *B. subtilis* from TMD led to higher excretion of *Bacillus* species in the feces, while the levels of *Akkermencia* were relatively lower in fecal bacteria. TMD in *B. subtilis* might modulate other gut bacteria, including *Bacillus* species, leading to altering the fecal bacteria. HSLA and HSHA did not increase *Bacillus* species in the feces, suggesting that *B. subtilis* and other *Bacillus* species might increase in the gut. Interestingly, *B. subtilis* was one of the primary fecal bacteria in the LSLA group, indicating that *B. subtilis* in the LSLA might not reside in the gut. Additionally, TMD intake increased not only the abundance of *Bacillus* species but also *Lactobacillus* and *Ligilactobacillus* species in the feces compared to the Control and CSB groups. Our previous study demonstrated that a comparable TMD intake resulted in an increase in *Akkermencia* but a decrease in *Ligilactobacillus* in the cecal bacteria of the HSLA and LSLA groups compared to the Control group. These findings highlight the differences in bacterial content between cecal and fecal samples after the intervention and emphasize the need to interpret fecal bacterial data carefully. Considering the findings from our previous and present studies, TMD exhibits synbiotic properties and shows potential for beneficial effects on bone metabolism.

Fermented foods can sometimes contain toxins produced by specific bacteria. Among the common toxins are mycotoxins, including aflatoxin B1, bacterial toxins such as botulinum and amylosin, as well as biogenic amines [44,45]. Aflatoxins, which Aspergillus generates, are heat-stable and remain unaffected by ordinary cooking methods. Reducing aflatoxin levels in fermented foods is important, and certain strains of *B. subtilis*, *B. amyloliquefaciens*, and *B. lincheniformis* have been found to degrade aflatoxins effectively [45,46,47]. Considering TMD, which contains a high amount of *B. subtilis*, the strains in the TMD can help degrade aflatoxins during fermentation, thus reducing their presence in the final product. However, it should be noted that certain *B. amyloliquefaciens* strains have been reported to produce amylosin, a heat-stable compound that can stimulate cytokines from human macrophages [46]. Biogenic amines, such as histamine and tyramine, present in some fermented foods, can potentially lead to adverse effects [18,19]. Nevertheless, some strains of *B. subtilis* have the ability to degrade these biogenic amines during the fermentation process, which contributes to improved food safety. The present study investigated the levels of biogenic amines in TMD enriched with *B. subtilis* and found that they did not induce harmful effects. It suggests that the presence of *B. subtilis* might contribute to reducing the production of certain toxins during fermentation. However, further research is required to isolate specific strains of *B. subtilis* that can effectively reduce toxins during the fermentation of TMD. 

There were some limitations of the present study. (1) The study used ovariectomized (OVX) rats as a model for menopausal women. While animal models provide valuable insights, there may be differences in responses between animals and humans, limiting the direct applicability of the findings to human populations. (2) The intervention period was limited to 12 weeks. Long-term effects and safety considerations of prolonged TMD consumption were not explored, and additional studies with extended follow-up periods are necessary. (3) The study suggests potential benefits of *B. subtilis*-rich TMD for menopausal women. However, this study did not establish specific dosages and optimal formulations of TMD for human consumption, warranting further investigation. (4) The study relied on an animal model, and extrapolating the results to human populations requires caution. 

In conclusion, menopause is characterized by typical menopausal symptoms and insulin resistance. Exacerbation of insulin resistance during menopause leads to the dysregulation of glucose metabolism, bone loss, and memory dysfunction. This study aimed to examine the potential of TMD intake to modulate glucose and bone homeostasis and memory function in OVX rats. The findings revealed that consuming different types of TMD, distinguished by the abundance of *B. subtilis* and biogenic amines, resulted in improved insulin resistance, reduced serum glucose concentrations, and mitigated memory decline compared to the Control group. Notably, HSHA and HSLA formulations exhibited the ability to preserve BMD and counteract markers of osteoclastic activity. However, it is essential to acknowledge that the BMD of the TMD-treated groups did not reach the levels observed in the Normal-control group. Consequently, TMD enriched with high *B. subtilis* content may be recommended for enhancing glucose metabolism and partially improving BMD and memory function. More long-term animal studies to examine the effects over extended periods would offer valuable insights into the potential sustainability and long-lasting benefits of incorporating TMD into the diet. Further randomized clinical trials with a large number of postmenopausal women are warranted to validate the potential benefits of TMD and to provide evidence-based recommendations.

## Figures and Tables

**Table 1:** Characteristics of dried traditionally-made doenjang (TMD).

	LSHA	HSHA	HSLA	LSLA	CSB
Sodium (%)	8.8 ± 0.1 ^a^	7.4 ± 0.2 ^c^	9.0 ± 0.1 ^a^	8.5 ± 0.2 ^b^	-
Water (%)	56.7 ± 0.37 ^b^	59.2 ± 0.32 ^a^	51.9 ± 0.33 ^c^	49.9 ± 0.36 ^d^	
Histamine (ug/g)	796 ± 1.4 ^b^	954 ± 0.6 ^a^	22.7 ± 0.2 ^d^	59.9 ± 0.4 ^c^	-
Tyramine (ug/g)	2629 ± 1.4 ^a^	1653 ± 1.4 ^b^	36.1 ± 1.4 ^d^	279 ± 1.7 ^c^	-
*Bacillus subtilis* (%)	15.0 ± 0.6	86.5 ± 2.3	92.0 ± 1.6	59.4 ± 1.5	-
*Bacillus licheniformis* (%)	35.1 ± 0.72	0.60 ± 0.0	0 ± 0	19.7 ± 0.12	-
*Weissella confuse* (%)	0.02 ± 0	4.3 ± 0.2	0.35 ± 0.01	0.01 ± 0	-
*Pediococcus acidilactici* (%)	0.16 ± 0	2.06 ± 0.07	0.05 ± 0	0.02 ± 0	-
*Bacillus coagulans* (%)	0.76 ± 0.02	0 ± 0	0 ± 0	0.03 ± 0	-
*Leuconostoc mesenteroides* (%)	0 ± 0	0.9 ± 0.04	0.01 ± 0	0.15 ± 0	-
*Leuconostoc citreum* (%)	0 ± 0	0.39 ± 0.01	0 ± 0	0.01 ± 0	-
*Staphylococcus aureus* (%)	0.09 ± 0	0 ± 0	0 ± 0	0.01 ± 0	-
*Acinetobacter baumannii* (%)	0.01 ± 0	0.04 ± 0	0 ± 0	0.01 ± 0	-
Daidzein (ug/g)	25.9 ± 0.05 ^b^	26.4 ± 0.62 ^b^	29.9 ± 0.48 ^a^	13.4 ± 0.31 ^c^	8.4 ± 0.11 ^d^
Genistein (ug/g)	43.8 ± 0.47 ^b^	42.6 ± 0.54 ^b^	47.2 ± 0.34 ^a^	26.3 ± 0.38 ^c^	9.2 ± 0.09 ^b^
Glycitein (ug/g)	6.7 ± 0.11 ^a^	6.3 ± 0.08 ^a^	6.8 ± 0.9 ^a^	1.8 ± 0.22 ^b^	3.5 ± 0.17 ^ab^
Daidzin (ug/g)	-	-	-	1.2 ± 0.09	35.4 ± 0.39
Genistin (ug/g)	-	-	-	2.5 ± 0.08	50.5 ± 0.58
Glycitin (ug/g)	-	-	-	-	8.7 ± 0.24
Total isoflavonoid aglycans (ug/g)	76.4 ± 1.87 ^b^	75.3 ± 1.11 ^b^	83.9 ± 0.73 ^b^	45.2 ± 0.56 ^c^	116 ± 0.82 ^a^

Values represented means ± standard deviation (*n* = 5). HSLA, TMD with high contents of *Bacillus subtilis (B. subtilis)* and high biogenic amines. HSHA, TMD with high contents of *B. subtilis* and low biogenic amines. LSHA, TMD with low contents of *B. subtilis* and high biogenic amines. LSLA, TMD with low contents of *B. subtilis* and low biogenic amines. ^a,b,c,d^ Different letters on the bars indicate a significant difference among the groups by Tukey’s test at *p* < 0.05.

**Table 2:** Serum glucose, insulin, and 17β-estradiol concentrations after 12-week intervention.

	Control	LSHA	HSHA	HSLA	LSLA	CSB	Normal-Con
Weight gain (g/12 week)	175 ± 12.1 ^a^	150 ± 12.7 ^b^	157 ± 10.1 ^b^	152 ± 12.1 ^b^	159 ± 14.2 ^b^	165 ± 16.3 ^ab^	117 ± 13.4 ^c^
Visceral fat (g)	11.1 ± 0.95 ^a^	10.8 ± 1.02 ^a^	7.73 ± 0.84 ^b^	7.35 ± 0.77 ^b^	5.92 ± 0.63 ^c^	7.15 ± 0.82 ^b^	5.75 ± 0.73 ^c^
Food intake (g/day)	13.6 ± 1.8	12.2 ± 1.3	12.6 ± 1.2	12.5 ± 1.4	13.0 ± 1.3	12.9 ± 1.4	11.6 ± 1.7
Uterine weight (g)	0.16 ± 0.02 ^b^	0.15 ± 0.02 ^b^	0.17 ± 0.03 ^b^	0.15 ± 0.02 ^b^	0.16 ± 0.02 ^b^	0.15 ± 0.02 ^b^	0.65 ± 0.03 ^a^
Serum 17β-estradiol(pg/mL)	1.56 ± 0.10 ^b^	1.52 ± 0.14 ^b^	1.61 ± 0.25 ^b^	1.49 ± 0.22 ^b^	1.53 ± 0.28 ^b^	1.42 ± 0.29 ^b^	7.47 ± 0.88 ^a^
Fasting serum glucose (mg/dL)	113 ± 4.47 ^a^	104 ± 5.58 ^ab^	103 ± 4.76 ^b^	102 ± 5.34 ^b^	109 ± 6.03 ^ab^	109 ± 7.46 ^ab^	95.3 ± 5.43 ^c^
2h-postprandial serum glucose (mg/dL)	143 ± 7.23 ^a^	126 ± 6.75 ^b^	126 ± 6.55 ^b^	126 ± 7.56 ^b^	141 ± 7.32 ^a^	135 ± 7.44 ^ab^	125 ± 6.13 ^b^
Fasting plasma insulin (ng/mL)	1.56 ± 0.19 ^a^	1.11 ± 0.19 ^b^	1.04 ± 0.13 ^b^	0.83 ± 0.19 ^c^	1.49 ± 0.21 ^a^	0.93 ± 0.19 ^bc^	0.86 ± 0.10 ^c^
HOMA-IR	6.53 ± 0.53 ^a^	4.93 ± 0.53 ^b^	3.97 ± 0.34 ^c^	3.11 ± 0.44 ^c^	6.02 ± 0.59 ^a^	3.75 ± 0.51 ^c^	3.04 ± 0.26 ^c^

Values represent mean ± standard deviation (*n* = 10). HOMA-IR, homeostasis model assessment estimate for assessing insulin resistance. HSLA, TMD with high contents of *Bacillus subtilis (B. subtilis)* and high biogenic amines. HSHA, TMD with high contents of *B. subtilis* and low biogenic amines. LSHA, TMD with low contents of *B. subtilis* and high biogenic amines. LSLA, TMD with low contents of *B. subtilis* and low biogenic amines. ^a,b,c^ Different letters in each variable indicate significant differences in one-way ANOVA at *p* < 0.05.

**Table 3:** Bone mineral density (BMD) of the femur by micro-CT after a 12-week intervention.

	Control	LSHA	HSHA	HSLA	LSLA	CSB	Normal-Con
BMD (g/cm^3^)	0.065 ± 0.016 ^d^	0.106 ± 0.013 ^c^	0.122 ± 0.016 ^c^	0.18 ± 0.047 ^b^	0.081 ± 0.019 ^d^	0.114 ± 0.001 ^c^	0.23 ± 0.016 ^a^
BV/TV (%)	11.4 ± 2.1 ^e^	15.9 ± 1 ^d^	17.9 ± 2.0 ^c^	23.8 ± 3.0 ^b^	13.5 ± 2.1 ^e^	17.1 ± 1.3 ^c^	30.6 ± 1.1 ^a^
Tb.Th (mm)	0.096 ± 0.003 ^c^	0.1 ± 0.005 ^b^	0.106 ± 0.002 ^a^	0.099 ± 0.001 ^b^	0.098 ± 0.004 ^b^	0.098 ± 0.003 ^b^	0.111 ± 0.003 ^a^
Tb.N (1/mm)	1.18 ± 0.08 ^c^	1.6 ± 0.18 ^b^	1.63 ± 0.19 ^b^	2.39 ± 0.36 ^a^	1.38 ± 0.24 ^c^	1.74 ± 0.01 ^b^	2.71 ± 0.08 ^a^
Tb.Sp (mm)	1.12 ± 0.08 ^a^	0.77 ± 0.19 ^b^	0.81 ± 0.15 ^b^	0.49 ± 0.21 ^c^	1.02 ± 0.23 ^a^	0.72 ± 0.05 ^b^	0.4 ± 0.08 ^c^

Values represent mean ± standard deviation (*n* = 10). HSLA, TMD with high contents of *Bacillus subtilis* (*B. subtilis)* and high biogenic amines. HSHA, TMD with high contents of *B. subtilis* and low biogenic amines. LSHA, TMD with low contents of *B. subtilis* and high biogenic amines. LSLA, TMD with low contents of *B. subtilis* and low biogenic amines. BMD, bone mineral density; BV/TV (%), bone volume/tissue volume; BS/BV, bone surface/bone volume; Tb.Th, trabecular thickness; Tb.N, trabecular number; Tb.Sp, trabecular separation. ^a,b,c,d,e^ Different letters in each variable indicate significant differences in one-way ANOVA at *p* < 0.05.

**Table 4:** Bone-metabolism-related parameters after the 12-week intervention.

	Control	LSHA	HSHA	HSLA	LSLA	CSB	Normal-Con
PTH (ng/mL)	43.9 ± 5.49 ^a^	38.8 ± 4.62 ^b^	28 ± 5.15 ^c^	32 ± 6.7 ^bc^	39.4 ± 5.03 ^ab^	31.4 ± 6.32 ^bc^	37.5 ± 3.89 ^b^
RANKL (pg/mL)	32.3 ± 3.43 ^a^	29.4 ± 3.15 ^b^	28.1 ± 3.52 ^b^	24.1 ± 3.12 ^c^	30.9 ± 3.73 ^ab^	27.9 ± 4.12 ^b^	22.4 ± 3.52 ^c^
OPG (ng/mL)	14.4 ± 0.95 ^c^	14.7 ± 0.97 ^c^	17.5 ± 0.83 ^b^	16.9 ± 0.95 ^b^	18.9 ± 1.17 ^a^	17.2 ± 0.94 ^b^	17.3 ± 0.91 ^b^
Osteocalcin (ng/mL)	1.59 ± 0.17 ^a^	1.40 ± 0.21 ^ab^	1.13 ± 0.18 ^b^	1.06 ± 0.17 ^b^	1.38 ± 0.15 ^ab^	1.05 ± 0.14 ^b^	0.69 ± 0.08 ^c^
BALP (U/L)	37.2 ± 4.58 ^a^	28.2 ± 4.72 ^b^	26.9 ± 3.68 ^b^	25.4 ± 5.09 ^b^	28 ± 5.01 ^b^	19.9 ± 4.18 ^c^	15.9 ± 3.2 ^c^

Values represent mean ± standard deviation (*n* = 10). HSLA, TMD with high contents of *Bacillus subtilis* (*B*.) *subtilis* and high biogenic amines. HSHA, TMD with high contents of *B. subtilis* and low biogenic amines. LSHA, TMD with low contents of *B. subtilis* and high biogenic amines. LSLA, TMD with low contents of *B. subtilis* and low biogenic amines. PTH, parathyroid hormone; OPG, Osteoprotegerin; RANKL, receptor activity of nuclear factor kappa B ligand; BALP, bone-specific alkaline phosphatase. ^a,b,c^ Different letters in each variable indicate significant differences in one-way ANOVA at *p* < 0.05.

## Data Availability

The data used to support the findings of this study can be made available by the corresponding author upon request.
